# The Influence of Matrix Resin Toughening on the Compressive Properties of Carbon Fiber Composites

**DOI:** 10.3390/polym16233328

**Published:** 2024-11-27

**Authors:** Xinfeng Ouyang, Xiao Wang, Qiufei Chen, Guojie Ge, Dong Liu, Kang Lin, Yunpeng Liu, Yangyang Zong, Shuo Duan, Kangmin Niu

**Affiliations:** 1 School of Materials Science and Engineering, University of Science and Technology Beijing, Beijing 100083, China; ouyangxf@ccgc.com.cn (X.O.); 15200096466@139.com (Y.L.); zongyangyang_001@163.com (Y.Z.); d18562122606@163.com (S.D.); 2 Zhongfu Shenying Carbon Fiber Company Limited, Lianyungang 222069, China; waxo0414@163.com (X.W.); qiufei_chen@126.com (Q.C.); gegj@zfsycf.com.cn (G.G.); liud@zfsycf.com.cn (D.L.); link@zfsycf.com.cn (K.L.)

**Keywords:** matrix resin, particle toughening, carbon fiber composites, compressive properties

## Abstract

The study investigated the effects of a toughening agent and micron-sized toughening particles (TP) on the resin and carbon fiber-reinforced polymer (CFRP) composites, with a particular focus on compressive strength. The results showed that the addition of the toughening agent improved the overall mechanical properties of both the resin and CFRP but had a minor effect on the residual compressive strength (CAI) of CFRP after impact. Compared to the pure toughening agent, the addition of TP increased the CAI, GIC, and GIIC of CFRP by 74%, 35%, and 68%, respectively. The SEM, ultrasonic C-scan, and metallographic microscopy were used to analyze the failure morphology and TP distribution. Compared to pure toughening agent modification, the introduction of TP led to the formation of continuous toughening particle layers, which reduced the compression damage area by 61%, significantly balancing and absorbing the load. This modification also resulted in typical kink band damage. This study found that resin toughening significantly improved the compressive strength of CFRP, while micron-sized toughening particles, in the form of toughening layers, notably improved the CAI. These findings provide valuable insights for enhancing the compression and impact resistance of CFRP.

## 1. Introduction

Carbon fiber composites are being increasingly applied in modern industrial systems, such as aerospace, hydrogen storage bottles, wind turbine blades, and new energy vehicles, due to their excellent mechanical properties and lightweight characteristics [1]. These materials improve application efficiency while reducing energy consumption and carbon emissions [2]. Carbon fiber composites are prepared by combining carbon fibers with matrix resins through specific processing techniques, with the matrix resin being one of the most important components of carbon fiber composites [3]. Under the condition that the interface performance satisfies the basic connection requirements between fibers and matrix resins, the matrix resin determines the fundamental mechanical properties of the fiber composites under the same fiber conditions. In China, efforts have been concentrated over the past few decades on solving the bottleneck technical challenges associated with carbon fibers [4]. Research on carbon fiber composites has primarily focused on fiber and interface studies, with an insufficient understanding of how matrix resins influence the overall performance of composites. The compressive strength of carbon fiber composites is significantly lower than their tensile strength, and their poor impact resistance largely restricts their application expansion. Enhancing the compressive strength and impact resistance of carbon fiber composites has become a challenge faced by the industry and a hot research topic [5,6,7,8,9]. Currently, the addition of nanomaterials to matrix resins is considered an effective approach to improving the compressive strength and impact resistance of carbon fiber composites [10]. Researchers have made positive progress in enhancing the compressive strength, impact resistance, and fracture toughness of carbon fiber composites by incorporating various nanomaterials, including rubber particles or carbon black [11,12,13,14], carbon nanotubes [15,16,17,18,19], graphene or graphene oxide [20,21,22,23,24], nano-clays [25,26,27,28], nano-silica [29,30,31,32,33,34], thermoplastic particles [35,36], and other nanostructures [37,38,39,40]. However, the main limitation of these studies is that they are often conducted at the laboratory level, focusing on one or several properties of composites. These investigations are not sufficiently systematic and present challenges in material selection and process design, thereby failing to provide direct references for industrial applications.

In this study, a toughened resin and micrometer-sized toughening particles were prepared to investigate the effects of resin toughening with toughening agents and subsequent particle toughening on the mechanical properties, particularly the compressive performance of carbon fiber composites. Unlike the majority of existing studies that focus on nanoparticle toughening, this research innovatively introduces micrometer-sized toughening particles that are significantly larger than the fiber diameter. By comparing the resin in its non-toughened state with the resin toughened by toughening agents, it was clearly demonstrated that the improvement in the compressive performance of carbon fiber composites is primarily related to the matrix resin. Toughening the matrix resin with toughening agents can significantly enhance the compressive performance of carbon fiber composites. Further incorporation of micrometer-sized toughening particles significantly enhances the post-impact compressive performance and interlaminar fracture toughness of carbon fiber composites. Toughening the matrix resin with toughening agents notably improves the compressive performance of carbon fiber composites. Furthermore, the incorporation of micrometer-sized toughening particles substantially enhances the post-impact compressive performance and interlaminar fracture toughness of the composites. This study is grounded in industrial applications and provides a reference for addressing the industrial challenge of poor compressive performance in carbon fiber composites.

## 2. Materials and Experimental Procedure

### 2.1. Experimental Materials

The carbon fiber used in this experiment was commercial dry-jet wet-spun SYT55-12K carbon fiber produced by Zhongfu Shenying Carbon Fiber Co., Ltd. (No. 1-6, Jinqiao Road, Dapu Industrial Zone, Lianyungang City, China). The carbon fiber was prepared into prepreg on an industrial prepreg production line. A self-made toughening particle was designated as TP. The matrix resins used were the commercially available WP-5100 epoxy resin system from Wells Advanced Materials (Shanghai) Co., Ltd. (Building 2, No. 558, Boyuan Road, Jiading District, Shanghai, China), a self-made high-toughness epoxy resin system, and a self-made high-toughness epoxy resin system with TP toughening, which were designated as E1, E2, and E3, respectively. The prepregs were prepared into composite materials, and the resulting samples were designated as EC1, EC2, and EC3.

### 2.2. Experimental Equipment

The equipment for the prepreg production line was provided by Flourish International Co., Ltd. (Taiwan, China), model FHPM-02-1270. The prepreg cutting machine was supplied by Hangzhou Aike Technology Co., Ltd. (Hangzhou, China), model BK3C2513. The autoclave was manufactured by Shandong Zhonghang Taida Composite Materials Co., Ltd. (Yantai, China), model R2021-0011. The tensile testing machine was produced by Shimadzu Corporation(Nishinokyo-Kuwabara-cho, Nakagyo-ku, Kyoto, Japan), model AG-XPLUS. The precision engraving milling machine was supplied by QiaoKe CNC Machinery Equipment Co., Ltd. (Jinan, China), model QK6090-1. The hot air circulation drying oven was provided by Suzhou Deruipu Oven Manufacturing Co., Ltd.(Suzhou, China), model DRP-8803. The vernier caliper was manufactured by SATA Tools (Shanghai) Co., Ltd. (Shanghai, China), model SATA 91511. The toughening resin and toughening particles were prepared using self-developed pilot-scale equipment.

### 2.3. Experimental Content

#### 2.3.1. Preparation of Toughening Particles

The specific details regarding the preparation of toughening particles involve trade secrets and will not be disclosed.

#### 2.3.2. Preparation of Toughened Resin Systems and Particle-Toughened Resin Systems

The specific preparation details of the toughened resin systems E2 and the particle-toughened resin systems E3 involving the aforementioned TP particles are considered proprietary and will not be disclosed.

**Figure 1:**
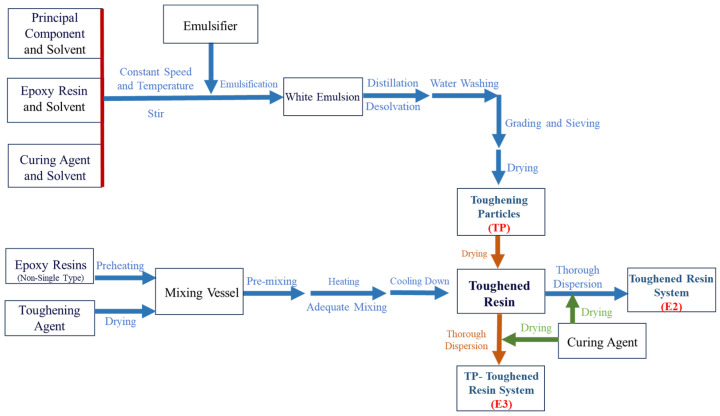
Process flow diagram for toughening particles (TP), toughened resin system (E2), and TP-toughened E2 (E3) resin systems.

The preparation process flow diagrams for toughening particles (TP), E2, and E3 are shown in Figure 1.

#### 2.3.3. Preparation of Prepregs and Composite Materials

**Figure 2:**
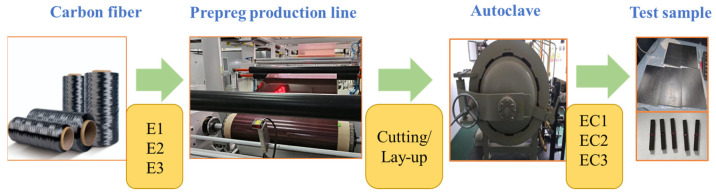
A simplified diagram for preparing composite material samples using carbon fiber prepreg.

The prepreg production line operates with 548 fibers, with a fiber areal density controlled at 194 g/m^2^ and a width maintained at 1270 mm. The mass content of resin is controlled at 34%, and the production line speed is set at 2 m per minute. The prepregs are cut according to the sizes and requirements specified in the testing standards. They are then manually stacked with the assistance of machinery based on the calculated number of layers. The stacked, pre-cured samples are placed into an autoclave and heated according to a programmed schedule for curing. The autoclave process is conducted as follows: the temperature is maintained at 70 °C for 70 min, followed by a heating rate of 1.5 °C/min to 130 °C, which is then held at 0.6 MPa for 130 min. Subsequently, the laminates are cut into the required sample sizes and quantities for testing standards using a precision milling machine. The flowchart for preparing prepreg from carbon fibers and then fabricating composite test samples using an autoclave is shown in Figure 2.

### 2.4. Characterization Methods

#### 2.4.1. Characterization of the Resin

(1) The mechanical properties of the resin are tested according to the GB/T 2567-2021 standard [41] for performance testing of resin castings, including tensile strength, flexural strength, and impact strength, the number of test samples is 6. The melting conditions are set to 140 °C for 2 h, and the curing conditions are set to 170 °C for 2 h, with the preparation process assisted by a degassing machine.

(2) The glass transition temperature (Tg) is tested using dynamic mechanical analysis (DMA). The TA Instruments DMA 850 is utilized, with conditions set between 40 °C and 280 °C, at a heating rate of 5 °C/min, and operated in the DMA multi-frequency strain mode at a frequency of 1.0 Hz, following the ASTM E1545-2011 [42] standard test method for the glass transition temperature of polymers via thermomechanical analysis. The storage modulus (E’) reflects the elastic characteristics of the sample and indicates its ability to fully recover from deformation. The loss modulus (E”) represents the viscous characteristics of the sample, indicating the heat loss during deformation. The loss factor (tanδ), defined as the ratio of the loss modulus to the storage modulus, reflects the vibration absorption capability.

#### 2.4.2. Characterization of Carbon Fibers

The testing process is controlled at an ambient temperature of 25 °C ± 1 °C and relative humidity below 45%. The linear density, tensile strength, Young’s modulus, and elongation at break of the carbon fibers are measured according to the GB/T 3362-2017 standard [43]. The resin content of the fibers is determined using the Soxhlet extraction method specified in GB/T 29761-2022 [44] Method A.

#### 2.4.3. Characterization of Carbon Fiber Composites

(1) The tensile strength and tensile modulus at 0° were determined according to ASTM D3039/D3039M-2014 [45], which specifies standard testing methods for the tensile properties of polymer matrix composites.

(2) The flexural strength and flexural modulus at 0° were measured in accordance with ASTM D7264/D7264M-2015 [46], which outlines the standard test methods for the flexural properties of polymer matrix composites.

(3) The compressive strength and compressive modulus at 0° were evaluated according to SACMA SRM 1R-94 [47], which specifies the testing method for the compressive properties of oriented fiber–resin composites.

(4) The compressive strength after impact (CAI) was assessed in accordance with ASTM 7136/D7136M-20 [48], which measures the damage resistance of fiber-reinforced polymer matrix composites to drop impact events, combined with ASTM D7137/D7137M-17 [49], which details the standard testing method for the residual compressive strength characteristics of damaged polymer matrix composite plates. An impact head with a diameter of 16 mm was utilized, with the impact energy calibrated to 6.7 J/mm. The dimensions of the specimen were 150 mm × 100 mm × 4.5 mm, leading to an impact energy calculation of 4.5 mm × 6.7 J/mm = 30.15 J.

(5) The open-hole tensile strength (OHT) was determined according to ASTM D5766/D5766M-2011 [50], which provides the standard test method for the open-hole tensile strength of polymer matrix composite laminates. The dimensions of the open-hole tensile specimen were 300 mm × 36 mm × 3 mm with a hole diameter of 6 mm.

(6) The open-hole compressive strength (OHC) was measured according to D6484/D6484M-2014 [51], which outlines the standard test method for the open-hole compressive strength of polymer matrix composite laminates.

(7) The interlaminar shear strength (ILSS) was determined according to ASTM D2344/D2344M-2016 [52], which provides the standard test method for the short-beam shear strength of polymer matrix composites and their laminates.

(8) The mode I interlaminar fracture toughness (G_IC_) was assessed in accordance with ASTM D 5528/D 5528M-2013 [53], which specifies the standard test method for the mode I interlaminar fracture toughness of unidirectional fiber-reinforced polymer matrix composites.

(9) The mode II interlaminar fracture toughness (G_IIC_) was evaluated using the ASTMD7905/D7905M-2014 [54] testing method, which measures the mode II interlaminar fracture toughness of unidirectional carbon fiber reinforced polymer matrix composites under mode II shear loading using edge-notched beam specimens.

(10) A C-scan was performed on the composite plates subjected to an impact energy of 30.15 J using the NBYSH-C011 ultrasonic flaw detector, following the method outlined in ASTM D7136/D7136M-20 [48].

(11) The morphology of the compression fracture surfaces of the carbon fiber composites was observed under a Hitachi REGULUS series SU8100 (Hitachi limited, Tokyo, Japan) field emission scanning electron microscope.

(12) The distribution of toughening particles within the prepreg was examined using a Leica DM2700M upright metallurgical microscope(Leica Microsystems, Wetzlar, Germany).

## 3. Results and Discussion

### 3.1. Characterization of Resin Properties

#### 3.1.1. Mechanical Properties of the Resins

**Figure 3:**
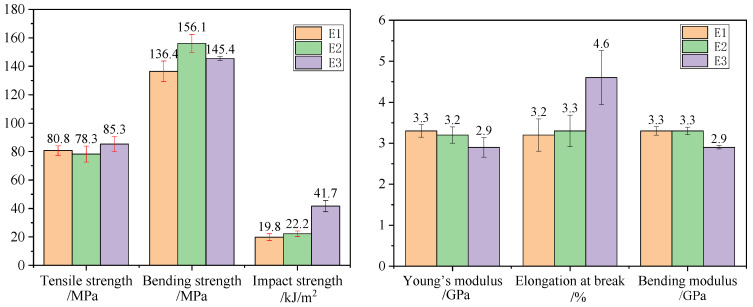
Comparison of mechanical properties of three resin systems.

The mechanical properties of the three resin systems are summarized in Table 1, while Figure 3 shows the comparison of mechanical properties of three resin systems. The tensile strength of the three resin systems is approximately 80 MPa. The addition of toughening particles resulted in an approximately 9% increase in the tensile strength of E3, indicating that the toughening particles allow E3 to bear tensile loads more evenly and distribute them better, with the interpenetrating structure contributing to enhanced strength. The bending strengths of the toughened E2 and E3 resins increased by approximately 14% and 7%, respectively, compared to E1. Toughening resulted in a decrease in the modulus of the resin systems; the addition of toughening particles led to further reductions in the tensile and bending moduli of E1, E2, and E3, which exhibited a downward trend. The incorporation of toughening agents and particles led to a certain reduction in the stiffness of the resin systems, resulting in lowered modulus performance while the elongation at break increased. E3 showed an increase of 1.3 percentage points in elongation at break compared to E2. Toughening enhanced the impact strength of the resins, with the toughening particles significantly contributing to this improvement. The impact strength of E2 increased by 12% compared to E1, while E3 improved by 110% and 87% compared to E1 and E2, respectively.

#### 3.1.2. Thermodynamic Properties of the Resins

**Figure 4:**
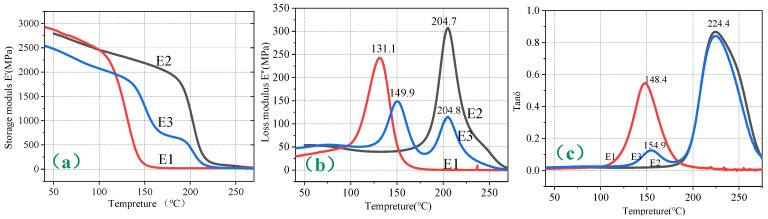
DMA plots of three resin systems ((**a**) storage modulus, (**b**) loss modulus, (**c**) loss factor).

From Figure 4, it can be seen that the storage modulus (E1) starts to decline at a relatively low temperature and at a rapid rate. The loss modulus curve exhibits a distinct single peak, as E1 is a medium-temperature resin system. The inherent properties of the resin determine its glass transition temperature (Tg) to be around 130 °C; therefore, the storage modulus begins to decrease rapidly at approximately 110 °C. Conversely, E2 and E3 are high-temperature systems; the storage modulus of the toughened E2 begins to drop rapidly around 180 °C and stabilizes thereafter. The storage modulus curve of E3 exhibits dual platforms due to the incorporation of toughening particles, with the low-temperature platform commencing a decline around 145 °C, indicative of the toughening particles, and the high-temperature platform beginning to drop around 180 °C, reflective of the resin. Both the loss modulus and loss factor curves display dual peaks, indicating poor compatibility between the toughening particles and resin, thus suggesting the existence of a biphasic structure. The DMA spectra indicate three methods for determining the glass transition temperature (Tg): the onset temperature of the storage modulus curve, and the peak temperatures of the loss modulus and loss factor curves, with these temperatures progressively increasing. According to ISO standards, it is recommended to use the peak temperature of the loss modulus as the Tg. The Tg values determined from the loss modulus (E”) peak temperatures for E1, E2, and E3 were 131.1 °C, 204.7 °C, and 149.9 °C/204.8 °C, respectively. Detailed DMA data are shown in Table 2.

### 3.2. Effect of Toughened Matrix Resin on the Compressive Properties of Carbon Fiber Composites

#### 3.2.1. Mechanical Properties of Carbon Fiber

The carbon fiber used in this batch of experiments was produced from the same batch on the production line. The mechanical property data of the carbon fiber is providedin Table 3:

#### 3.2.2. Mechanical Properties Analysis of Carbon Fiber Composites

The mechanical properties of each composite sample were tested following the aforementioned methods, with the data results presented in the Table 4.

**Figure 5:**
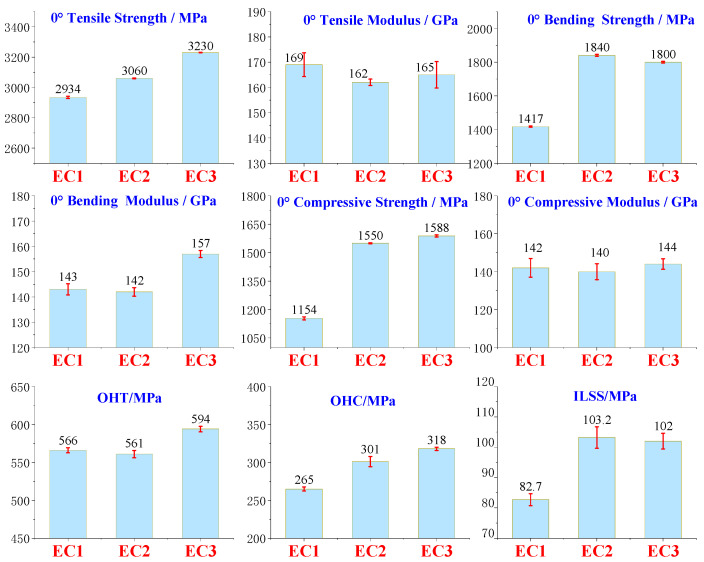
Mechanical properties test results of composite materials.

By comparing EC2 and EC1, it was found that the toughening of the matrix resin had a minor effect on the tensile strength and modulus of the carbon fiber composites, as the 0° tensile strength, open hole tensile strength, 0° tensile modulus, 0° bending modulus, and 0° compressive modulus showed slight decreases, maintaining an overall similar level. This indicates that the toughening agents used to enhance the matrix resin do not significantly contribute to the tensile strength and modulus metrics of the carbon fiber composite system, as the carbon fiber or the carbon fiber composite system’s properties predominantly govern these indicators. However, EC2 exhibited substantial improvements compared to EC1 in 0° bending strength, 0° compressive strength, open-hole compressive strength, and interlaminar shear strength, with increases of 30%, 34%, 14%, and 25%, respectively. The trend of the 0° bending strength enhancement in EC2 is consistent with that of E2 compared to E1. The increased toughness of the resin matrix positively contributed to the bending and compressive strength improvements of the carbon fiber composites. The toughened E2 system incorporates high-functionality epoxy, enhancing the interaction between the resin matrix and the active functional groups on the carbon fiber surface [55]^,^ which significantly improves the interlaminar shear strength. Additionally, the toughening agent reduces the brittleness of the resin, improves impact resistance, and enhances the ability to inhibit crack initiation and propagation, significantly improving the compressive strength of the carbon fiber composites. Furthermore, a comparison of the interlaminar fracture toughness (Mode I and Mode II) showed that EC2 improved by 15% and 24%, respectively, indicating a significant enhancement in interlaminar fracture toughness due to the toughening of the matrix resin, as shown in Figure 5. The CAI values for EC1 and EC2 differed by only 4 MPa, indicating that the toughening of the matrix resin was insufficient to enhance the impact performance of the carbon fiber composites under the conditions of 6.7 J/mm.

EC3 was developed based on the toughened resin of EC2, with the addition of toughening particles. Comparisons indicate that EC3 exhibited slight improvements over EC2 in the 0° tensile strength, 0° flexural modulus, open-hole tensile strength, and open-hole compressive strength, with increases of 6%, 11%, 6%, and 6%, respectively. This suggests that the toughening particles played a significant role in inhibiting crack initiation and rapid propagation within the composite material system, particularly reflected in the tensile strength of carbon fiber composites, which is predominantly governed by the intrinsic properties of the carbon fiber itself. The parameters of 0° tensile modulus, 0° flexural strength, 0° compressive strength, 0° compressive modulus, and interlaminar shear strength remained at a consistent level, indicating that further toughening with particles did not contribute positively to these performance metrics.

**Figure 6:**
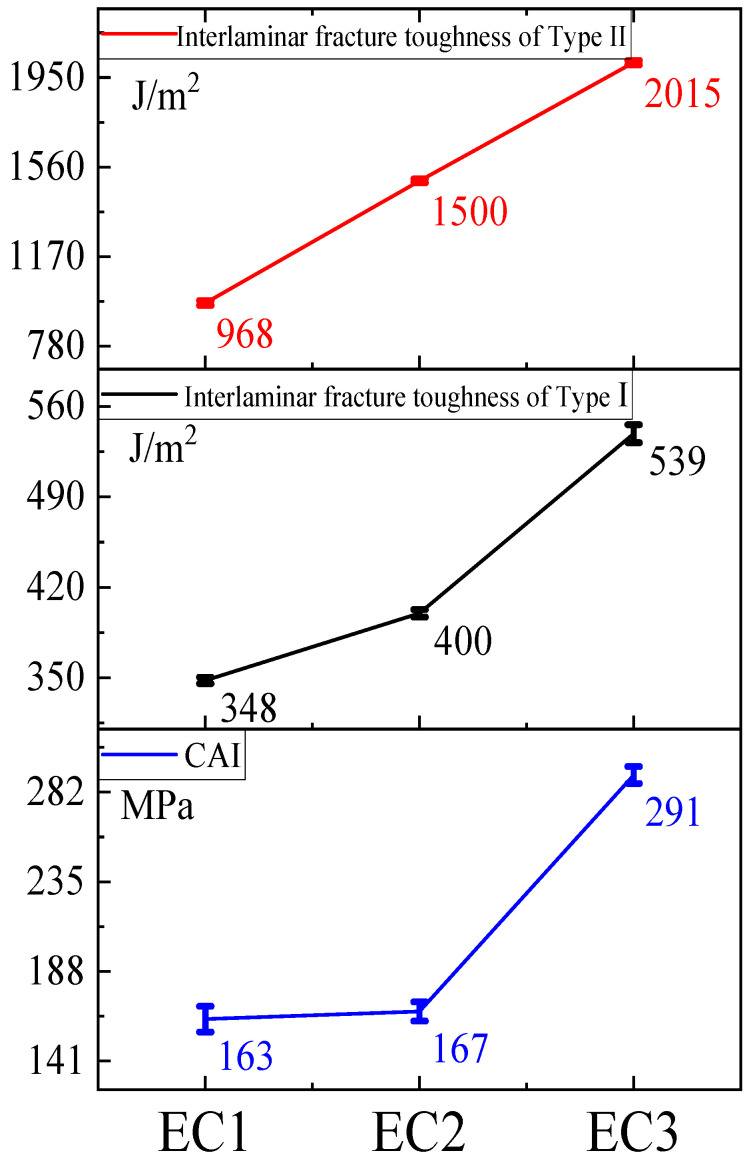
Results of G_ⅠC_, G_ⅡC_, and CAI for carbon fiber composites.

Further comparisons of the interlaminar fracture toughness in Mode I and Mode II, as well as the Compression After Impact (CAI) index, as shown in Figure 6, revealed that EC3 demonstrated increases of 35%, 68%, and 74%, respectively, compared to EC2. The toughening particles exhibited exceptional performance in enhancing the impact resistance of carbon fiber composites. Under external forces, these particles effectively inhibited the generation and propagation of cracks, allowing the composite material to withstand greater external forces. This was particularly notable in the areas of post-impact compressive strength and interlaminar fracture toughness. When subjected to impact forces, cracks expanding through the interlaminar regions were obstructed by the fine particles, enabling elastic deformation to absorb impact energy, thereby reducing the driving force for further crack propagation and significantly improving the CAI.

A comprehensive comparison among EC1, EC2, and EC3 indicated that the use of toughening agents in the matrix resin improved the 0° compressive strength of carbon fiber composites by 34%, although there was limited enhancement in post-impact compressive strength. On this basis, the further addition of toughening particles led to a 74% increase in post-impact compressive strength, while the further enhancement of 0° compressive strength was minimal. The toughening agents significantly improved the interlaminar fracture toughness in both G_IC_ and G_IIC_ modes, and further particle toughening resulted in substantial increases in both G_IC_ and G_IIC_. It was concluded that the toughening of the matrix resin contributed significantly to the enhancement of compressive strength in carbon fiber composites, whereas the particle toughening made a notable contribution to improving the post-impact compressive performance of the composites.

#### 3.2.3. Microstructural State of Particle Toughened Carbon Fiber Composites

**Figure 7:**
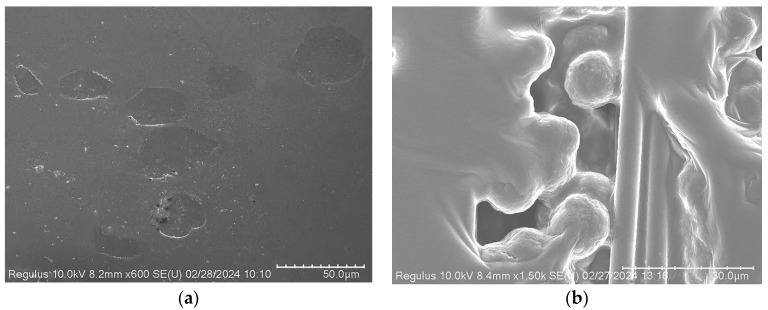
High-resolution electron microscopy images of toughening particles in (**a**) E3 and (**b**) EC3.

**Figure 8:**
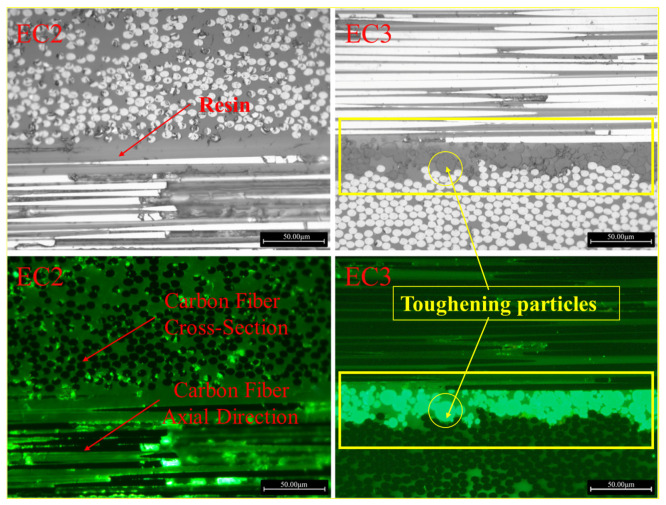
Metallographic comparison of EC2 and EC3 with and without toughening particles.

The E3 resin was dried, and the distribution state of the toughening particles in the EC3 samples was observed using a high-resolution electron microscope, as shown in Figure 7a,b. The average size of the toughening particles was approximately 30 μm, significantly larger than the carbon fiber diameter (about 5 μm), exhibiting elliptical or nearly circular shapes with good dispersion within the resin. Further observations of the EC2 and EC3 samples were conducted using metallographic microscopy under normal and fluorescent conditions, as shown in Figure 8. The images in the right column reveal a significant distribution of toughening particles in the resin layer between the prepreg layers of the carbon fiber composites, indicating a nearly continuous phase distribution that formed a continuous micrometer-scale layer of toughening particles between the fiber layers. In Figure 8, both the cross-sections (circular) of carbon fibers and their states along the axial direction can be observed, which is due to the lamination of the composite samples with 0° and 90° orientations during the preparation according to the testing standards.

#### 3.2.4. Mechanism of Particle Toughening and Compression Failure Morphology

(1) Post-Impact C-Scan Images of Carbon Fiber Composite Samples

**Figure 9:**
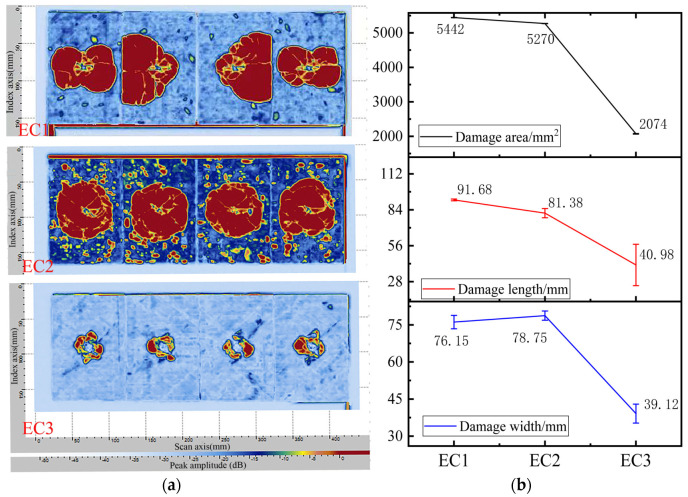
C-Scan images of carbon fiber composites after impact (**a**) and processing results (**b**).

The composite material samples were prepared according to the post-impact compressive strength testing method and subjected to an impact energy of 30.15 J, followed by ultrasonic C-scan analysis, as presented in Figure 9a. The damage area, damage length, and damage width data are displayed in Figure 9b. As seen in Figure 9a, in the case of EC1, two of the four samples exhibited damage areas exceeding the boundary of the composite test panel following impact, indicating widespread crack propagation and substantial damage area, thus demonstrating poor impact resistance. Although the damage area for the toughened EC2 was also considerable, the impacted regions remained within the boundaries of the composite test panel, effectively restraining crack propagation. In contrast, the particle-toughened EC3 exhibited a significantly reduced damage area following impact, reflecting a marked improvement in impact performance. The data in Figure 9b reveal that the damage area for EC1 was determined based on the average of the two samples exhibiting the worst damage states, while the damage area for EC3 decreased by 61% compared to EC2 and by 62% compared to EC1. The incorporation of micron-sized toughening particles significantly enhanced the impact resistance of carbon fiber composites, effectively dispersing loads and controlling crack propagation within the composite material system. This created a robust interlaminar toughening mechanism characterized by micron-sized particles.

**Figure 10:**
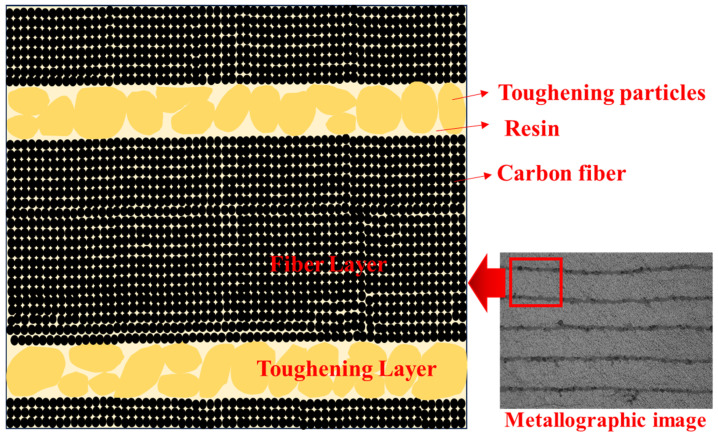
Model of micron-level particle interlaminar toughened carbon fiber composites.

Based on the analytical results and metallographic images, a toughening model for the formation of interlaminar toughening by micron-sized particles is illustrated in Figure 10. The fiber layers and toughening layers are distinctly identified, with the resin serving as the continuous matrix phase. Both the fibers and toughening particles are distributed within the resin phase, with the toughening layers interspersed between the fiber layers. Upon application of external impact forces on the composite, the toughening layers are capable of absorbing impact energy and facilitating load transfer and dispersion, effectively inhibiting crack propagation.

(2) SEM Images of 0° Compression Failure

**Figure 11:**
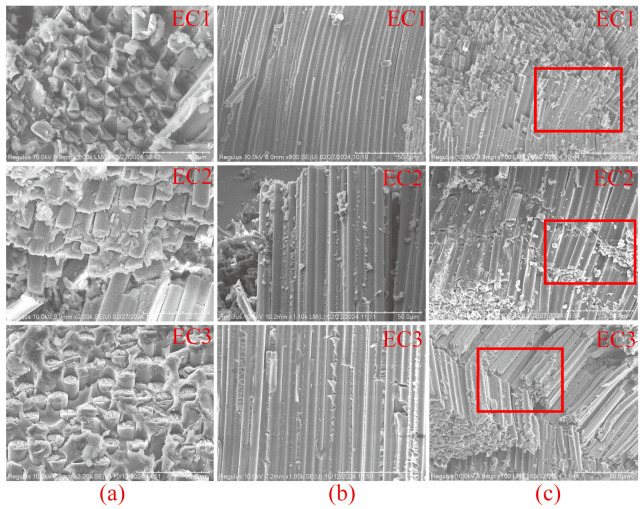
SEM morphology of compression failure in carbon fiber composites: (**a**) fracture end face, (**b**) fracture cross-section, (**c**) fiber fracture morphology distribution.

High-resolution scanning electron microscopy (SEM) analysis was conducted on the samples of carbon fiber composites that failed during the 0° compression strength test, as shown in Figure 11. Images (a), (b), and (c) represent the fracture end face, fracture cross-section, and fiber fracture morphology distribution, respectively. From Figure 11a,b, it can be observed that the end face exhibits no significant cracks, voids, or other defects, indicating that the bonding between the fibers and the resin is satisfactory across all three sample groups. This suggests that the interfacial condition between the carbon fibers and the resin meets the fundamental requirements for carbon fiber composites, and interfacial delamination [56] does not serve as a failure mechanism for compression fracture.

From the comparative analysis of the fiber distribution states after failure in carbon fiber composites, as shown in Figure 11c, it was found that the fiber fracture state in EC1(c) is characterized by fragmentation, with breakage occurring in small segments. This suggests that the matrix resin lacks the toughness to withstand compressive loads. Although the fibers in EC2(c) did not exhibit fragmentation, the length of the fractured fibers increased significantly, displaying characteristics of twisting band models of fracture. This indicates that the matrix resin plays a role in load distribution under compressive loads, effectively resisting the compressive loads. In EC3(c), features of breakage within the twisting band are evident, with fibers fracturing into longer segments, demonstrating that the toughness of the resin aids in resisting and absorbing compressive loads, leading to more distributed load-bearing by the fibers. The higher compressive strengths observed in EC2 and EC3 compared to EC1 confirm this behavior.

**Figure 12:**
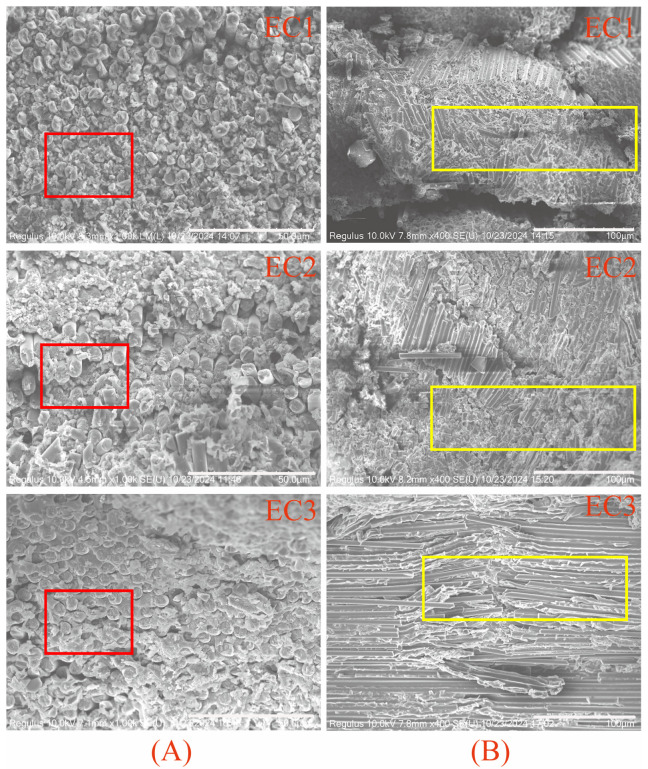
CAI failure SEM morphology of carbon fiber composites: (**A**) cross-sectional, (**B**) fiber radial distribution.

Further SEM observations were conducted on the failure morphologies of samples subjected to compression failure after impact, as shown in Figure 12. A comparison of Figure 12A reveals that the carbon fiber composites EC1 and EC2 exhibited significant resin phase failure after being subjected to impact loads. This indicates that the resin undergoes catastrophic failure instantaneously upon impact, resulting in concurrent fiber fracture with distinct pulverization characteristics. After sustaining impact loads, these composites have already incurred damage, making them unable to withstand subsequent compressive loads, as evidenced by the pronounced fiber fragmentation. In the EC3image, which has undergone particle toughening, the resin does not display the pulverization characteristics observed in EC1 and EC2. Neither the fibers nor the resin phase exhibit pulverization or delamination. Referring to Figure 12B, the EC1 composite shows a more fragmented fracture pattern, while the EC2) exhibits a more orderly fragmentation, characterized by kink band failure. The EC3 composite maintains good integrity of both fibers and resin, with clear kink band failure characteristics. The micron-level particle interlayer toughening layer effectively absorbs and distributes the loads during impact, allowing the composite system to remain intact and continue to withstand compressive loads, exhibiting a kink band failure mode. This is consistent with the significant improvement observed in the compressive after-impact performance (CAI).

## 4. Conclusions

This study focuses on resin toughening, with an emphasis on comparing the effects and mechanisms of toughening agents and toughening particles on the properties of carbon fiber composite materials:

(1) The pure toughening agent can significantly enhance the overall mechanical properties of the composite material, with a 34% increase in compressive strength, but its effect on improving the post-impact residual compressive strength (CAI) is limited.

(2) Compared to the pure toughening agent, the addition of micron-sized toughening particles (TP) increased the CAI, GIC, and GIIC of CFRP by 74%, 35%, and 68%, respectively. Meanwhile, the damage area was reduced by 61%.

(3) This research, set against the backdrop of improving industrial applicability, demonstrates the importance of resin toughening for the compressive performance of composite materials. Enhancing the CAI requires the presence of toughening particles. Through comprehensive mechanical performance testing, this study provides guidance for the industrial application of micron-scale toughening particles.

## Figures and Tables

**Table 1:** Mechanical properties of three resin systems.

	E1	E1-CV/%	E2	E2-CV/%	E3	E3-CV/%
Tensile strength/MPa	80.8	4.1	78.3	7.2	85.3	6.3
Young’s modulus/GPa	3.3	4.6	3.2	6.2	2.9	8.2
Elongation at break/%	3.2	12.3	3.3	11.7	4.6	14.4
Bending strength/MPa	136.4	5.3	156.1	4.1	145.4	1.0
Bending modulus/GPa	3.3	3.1	3.3	2.8	2.9	1.4
Impact strength/kJ/m^2^	19.8	12.4	22.2	8.9	41.7	9.5

**Table 2:** Tg results of three resin systems (DMA).

	The Onset Temperature of the E’ Curve	The Peak Temperature of the E” Curve	The Peak Temperature of the Tanδ Curve
**E1**	128.0	131.1	148.4
**E2**	197.9	204.7	224.4
**E3**	141.4	149.9	154.9
	200.8	204.8	224.4

**Table 3:** Mechanical properties data of SYT55-12K carbon fiber.

	Linear Density mg/m	Density g/cm^3^	Sizing Agent Content/%	Tensile Strength/MPa	Young’s Modulus/GPa
Test value	453	1.7796	1.12	6089	297
CV/%	1.1	2.4	1.7	3.9	0.7

**Table 4:** Mechanical properties data of carbon fiber composites under three resin systems.

	EC1	EC2	EC3	EC1-CV/%	EC2-CV/%	EC3-CV/%
0° Tensile Strength/MPa	2934	3060	3230	7.6	2.1	1.5
0° Tensile Modulus/GPa	169	162	165	4.7	1.3	5.2
0° Flexural Strength/MPa	1417	1840	1800	4.2	5.6	4.7
0° Flexural Modulus/GPa	143	142	157	2.2	1.7	1.4
ILSS-RTD/MPa	82.7	103.2	102.0	2.0	3.5	2.6
0° Compressive Strength/MPa	1154	1550	1588	7.8	3.3	6.0
0° Compressive Modulus/GPa	142	140	144	4.9	4.2	2.8
OHT/MPa	566	561	594	3.4	4.7	3.7
OHC/MPa	265	301	318	2.7	6.7	2.1
CAI/MPa	163	167	291	6.8	5.0	4.4
G_ⅠC_ /J/m^2^	348	400	539	2.5	2.9	6.9
G_ⅡC_/J/m^2^	968	1200	2015	10.1	8.3	9.5

## Data Availability

The original contributions presented in the study are included in the article, further inquiries can be directed to the corresponding author.
